# In Vitro Gene Delivery in Retinal Pigment Epithelium Cells by Plasmid DNA-Wrapped Gold Nanoparticles

**DOI:** 10.3390/genes10040289

**Published:** 2019-04-09

**Authors:** Sònia Trigueros, Elena B. Domènech, Vasileios Toulis, Gemma Marfany

**Affiliations:** 1 Department of Zoology, University of Oxford, Oxford OX1 3PS, UK; 2 Departament de Genètica, Microbiologia i Estadística, Universitat de Barcelona, 08028 Barcelona, Spain; elenabdomenech@ub.edu (E.B.D.); vtoulis@ub.edu (V.T.); 3 CIBERER, ISCIII, Universitat de Barcelona, 08028 Barcelona, Spain; 4 Institute of Biomedicine (IBUB-IRSJD), Universitat de Barcelona, 08028 Barcelona, Spain

**Keywords:** gene therapy, gold nanoparticles, DNA-wrapped gold nanoparticles, ARPE-19 cells, retinal pigment epithelium, clathrin-coated vesicles, endosomal trafficking

## Abstract

Many rare diseases course with affectation of neurosensory organs. Among them, the neuroepithelial retina is very vulnerable due to constant light/oxidative stress, but it is also the most accessible and amenable to gene manipulation. Currently, gene addition therapies targeting retinal tissue (either photoreceptors or the retinal pigment epithelium), as a therapy for inherited retinal dystrophies, use adeno-associated virus (AAV)-based approaches. However, efficiency and safety of therapeutic strategies are relevant issues that are not always resolved in virus-based gene delivery and alternative methodologies should be explored. Based on our experience, we are currently assessing the novel physical properties at the nanoscale of inorganic gold nanoparticles for delivering genes to the retinal pigment epithelium (RPE) as a safe and efficient alternative approach. In this work, we present our preliminary results using DNA-wrapped gold nanoparticles (DNA-gold NPs) for successful in vitro gene delivery on human retinal pigment epithelium cell cultures, as a proof-of-principle to assess its feasibility for retina in vivo gene delivery. Our results show faster expression of a reporter gene in cells transfected with DNA-gold NPs compared to DNA-liposome complexes. Furthermore, we show that the DNA-gold NPs follow different uptake, internalization and intracellular vesicle trafficking routes compared to pristine NPs.

## 1. Introduction

The dysfunction and death of photoreceptors are the main cause of vision loss in inherited retinal diseases (IRDs), a group of mendelian rare disorders with high genetic and clinical heterogeneity. After more than 30 years of intense clinical and genetic studies to identify IRD genes, around 300 causative genes have been identified that cause the dysfunction of photoreceptors or alter the function of the adjacent retinal pigment epithelium (RPE), thus leading to the progressive attrition of photoreceptors [1,2].

From the clinical side and most relevant to patients, the main challenge is to devise effective treatments to halt the progression of the disease or regain visual capacity. Depending on the gene and type of mutation, different molecular approaches for therapy can be devised; but, at least for autosomal recessive retinal diseases, the most straightforward strategy is the restoration of a fully functional version of the protein via DNA-based gene therapy, even though gene delivery to fully differentiated and mature cells is not an easy task [3,4]. On the other hand, the eye is an excellent target for gene therapy since it is accessible, amenable to non-invasive examination, possesses a well-defined anatomy and is relatively immune privileged [3,4,5]. Ocular gene therapy for retinal disorders is starting to be developed and the first commercial gene therapy to treat a severe infantile genetic blindness (Leber congenital amaurosis caused by bi-allelic mutations in the *RPE65* gene) was approved last year. These and other ongoing therapies in different clinical trial phases are based on adeno-associated virus (AAV) vectors [6]. However, viral gene delivery also raises several concerns, such as small size capacity (maximum packaging size for 5 kb), high production costs, probability of immunogenicity and inflammatory responses and their invasive route of administration to the retina (subretinal microinjection), which have fostered basic research on non-viral vectors such as nanoparticles (NPs) for gene delivery.

NPs are highly customizable, and can be designed and optimized for cellular uptake, bypassing the degradative machinery of the cells and improving gene expression in the nucleus [7]. Although a priori NPs might yield lower transgene expression levels compared to viral vectors, they overcome most safety concerns of viral vectors, are cost-effective and easily customizable and, most relevant, have a large DNA capacity, critical for gene delivery of large ocular disease genes [4,8]. All these desirable characteristics make the study of NPs for gene delivery to ocular tissues (cornea, retina and retinal pigment epithelium (RPE)) a research priority in order to explore viable alternatives to viral vectors. Nanoparticles made from different materials that display distinct properties, such as inorganic NPs, liposomes, solid lipids and polymeric NPs, have potential use in retinal cells [3,4,9,10]. Some successful attempts using compact DNA polycationic nanoparticles for gene delivery into the retina of pre-clinical animal models have also been reported [11,12].

However, more basic research is still needed to provide a full toolbox of NPs for different uses in the retina, including gene therapy. For instance, liposomal-based NPs, particularly those with positive charges, show cytotoxicity at the large quantities required for efficient gene delivery [13]. The use of polyethilenimine (PEI)-coated NPs increases cellular uptake but PEI is also cytotoxic [3,9]. Gold NPs are mostly non-toxic, but cellular uptake strongly depends on their size and diameter. Several reports indicate that gold NPs ranging between 20 and 50 nm are non-toxic [10]. Indeed, NPs display different physicochemical properties depending on the size, shape, surface charge and hydrophilicity, and these parameters directly impact cellular uptake by different types of vesicular and non-vesicular entry pathways, thereby determining the endocytic route and the final destination of NPs. In gene delivery, the internalization of NPs through the endosomes and eventual fusion of late endosomes with lysosomes should be avoided since it involves the destruction of nucleic acids by lysosomal hydrolases before the genetic material reaches the nucleus [7,14]. Moreover, it has been shown that the lysosomal accumulation of inorganic NPs, elicited by the acidic conditions of the lysosomal cellular compartment, enhance surface instability and ion release. Intracellular ion release is responsible for the cascading events associated with nanoparticle-induced intracellular toxicity [15] and therefore, accumulation of NPs in lysosomes should be avoided.

In this work, we explored the feasibility of gene delivery in cultured differentiated RPE cells using DNA-wrapped gold NPs (DNA-gold NPs). In particular, we focused on cellular uptake and intracellular endosomal trafficking routes of pristine gold NPs and DNA-gold NPs, showing that a higher proportion of DNA-gold NPs (compared to pristine gold NPs) are internalized through clathrin-independent routes that do not end up in late endosomes, thereby avoiding lysosomal degradation. These uptake routes most probably allow faster gene delivery into the nucleus as measured by an early expression of the green fluorescent protein (GFP) reporter gene.

## 2. Materials and Methods

### 2.1. ARPE-19 Cell Culture and Differentiation Conditions

Human ARPE-19 cells, acquired from ATCC (CRL_2302), were cultured in 1:1 Dulbecco’s Modified Eagle’s Medium (DMEM) (ATCC, Manassas, VA, USA) and Ham’s F-12 Nutrient Mix (Life Technologies, Carlsbad, CA, USA) supplemented with 10% foetal bovine serum (FBS) (Life Technologies, Carlsbad, CA, USA) and 1% penicillin-streptomycin (Life Technologies, Carlsbad, CA, USA) in a 5% CO_2_ humidified chamber at 37 °C. For immunocytochemistry, human ARPE-19 cells (1.5 × 10^5^cells/well) were seeded in growth medium without antibiotics onto poly-L-lysine-coated coverslips in 24-well plates. To induce differentiation, ARPE-19 cells were deprived of FBS for 48 h and maintained in a 1:1 media of DMEM and Ham’s F-12 Nutrient Mix. Subsequently, 48 h post-differentiation, cells were transfected with either nanoparticles or liposomes. For dynasore treatment, the media was changed at 48 h of differentiation, cells were washed once in PBS 1×, and dynasore was added at 80 μM (final concentration) for 30 min. Cell medium was then changed and cells were immediately nanotransfected.

### 2.2. Lipofection

After 48 h of differentiation, cells were co-transfected with the pEGFP reporter vector (500 ng/well), using Lipotransfectine (Niborlab, Guillena, Spain) (DNA: Lipotransfectine ratio of 1:3) in differentiation medium without antibiotics. This medium was replaced 5 h post-transfection with differentiation medium with antibiotics. After 16 h or 48 h of transfection, cells were fixed with 4% PFA for 20 min at RT and used for immunocytochemistry.

### 2.3. Gold NP Production and Nanotransfection

Gold nanoparticles (40 nm) at OD 1, stabilized in sodium citrate, were purchased from Sigma-Aldrich (St. Louis, MO, USA) (S-741981). One hundred microliters of gold nanoparticles at OD1 were spun down and resuspended in 10 μL of Milli-Q water and mixed with 1 μL (0.250 μg/μL) of pEGFP plasmid DNA per well. Plasmid DNA-gold hybrid structures were produced by allowing conditions in which double-stranded DNA (dsDNA) loops wrap over the surface of the nanoparticle. The dsDNA-nanoparticle complexes made by this method have an excellent dispersion in different solutions from water to cell culture medium [16]. DNA-wrapped gold nanoparticles were introduced in each well with seeded cells. Photothermal plasmon resonance of the DNA-gold nanoparticles was activated by white light irradiation [17]. Cells were allowed to grow for either 2 h (for immunocytochemistry), or 16 h/48 h (for EGFP expression). For transfection efficiency studies, cells were allowed to grow for 4 h before medium was changed. The details of the wrapping reaction and cell-transfection are currently subject to a patent and published under standard free patent procedures [18].

### 2.4. Immunocytochemistry

After 2 h, 16 h or 48 h post-transfection with either pristine gold NPs, DNA-wrapped gold NPs or liposomes, cells were fixed with 4% PFA for 20 min at RT, washed in PBS (3 × 5 min), permeabilized in 0.2% Triton X-100 (St. Louis, MO, USA) in PBS (20 min at RT), and blocked for 1 h in 4% sheep serum in PBS. Primary antibodies were incubated overnight at 4 °C in a 1:200 dilution in blocking solution (rabbit anti-GFP, rabbit anti-EEA1, mouse anti-RAB7, all from Abcam, Cambridge, UK). After incubation, coverslips with cells were rinsed in PBS 1× (3 × 5 min), incubated with the corresponding secondary antibodies conjugated to either Alexa Fluor 488, 568 or 647 (Life Technologies, Grand Island, NY, USA) (1:400) at RT (1 h) in blocking solution, nuclei were stained with DAPI (Roche Diagnostics, Indianapolis, IN, USA) (1:1000), washed again in PBS 1× (3 × 5 min), mounted in Mowiol 4–88 (Merck, Darmstadt, Germany) and analysed by confocal microscopy and optical transmission microscopy (Zeiss LSM 880, Thornwood, NY, USA). To quantify GFP-positive cells, coverslips were visualized in a ZOE™ Fluorescent Cell Imager (Bio-Rad, Hercules, CA, USA).

### 2.5. Imaging and Statistical Analyses

Image analyses were performed using ImageJ (FIJI) software 1.52i. Stack images from five consecutive planes (0.46 μm of separation each) centred on the subcellular location with maximum vesicle intensity were retrieved, segmented in the different channels (clathrin, EEA1, RAB7 or NP), measured and analysed. In the case that the total concentration of the protein of interest had to be determined, the fluorescence threshold value was manually selected and converted into a binary mask. Then, the binary mask of each protein was converted to area values. In the case that the area of the nanoparticles had to be determined, the threshold value was restricted to the dark spots produced by the plasmon resonance of the nanoparticles. The binary mask of NPs was superimposed over the image of interest—for example, the mask of the NP field was superimposed over the image of the clathrin-positive vesicles’ signal. Then, the intersection area between the two selected channels generated a new image, of which the total intensity was determined. To calculate the percentage of NPs in each type of intracellular vesicles, the area of intersection image (e.g., NPs on clathrin-positive cells) was measured and compared to the total area covered by NPs. For statistical analysis, three independent replicates were performed per condition, and three representative different regions of interest (ROIs) per condition and replicate were quantified. Values, ratios and statistical significance were analysed using GraphPad Prism 7.03 (San Diego, CA, USA).

## 3. Results

### 3.1. Comparison of Transfection Efficiency of Standard Liposomes Versus DNA-Wrapped Gold NPs in Differentiated ARPE-19 Cells

**Figure 1:**
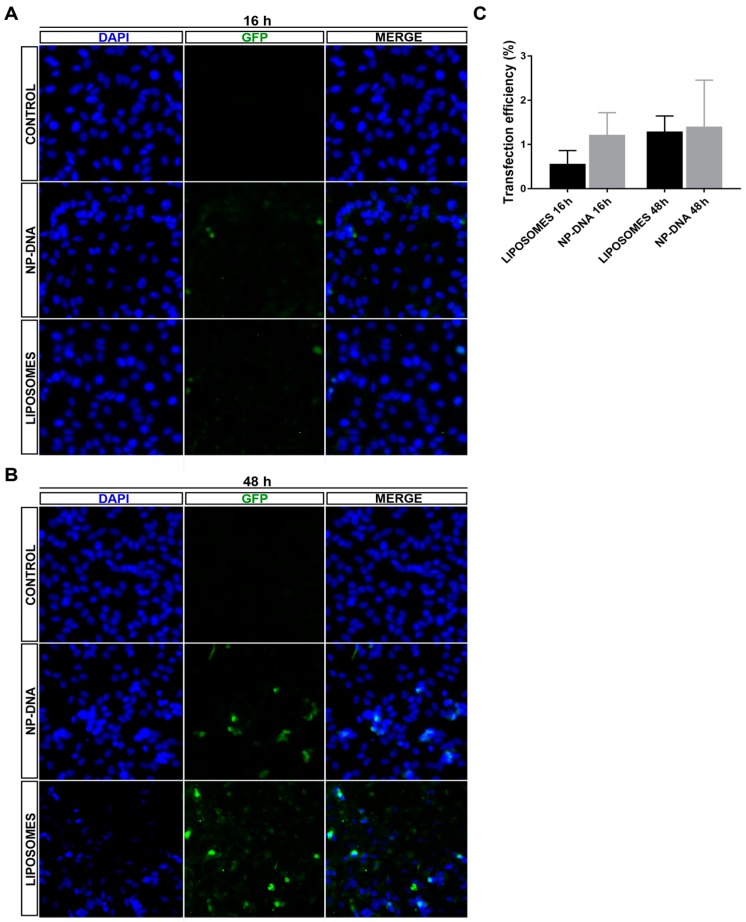
Analysis of transfection efficiency of DNA-wrapped gold nanoparticles (40 nm) compared to liposomes in differentiated ARPE-19 cells. Representative images of differentiated ARPE-19 cells transfected with the pEGFP reporter vector using either liposomes (LIPOTRANSFECTINE) or nanoparticles at (**A**) 16 h and (**B**) 48 h (for wider field images with a lower amplification, see Supplementary Materials, Figure S1). (**C**) Quantification of green fluorescent protein (GFP)-positive cells showed similar levels of transfection efficiency by nanoparticles (1.4% positive cells using 0.25 μg/150,000 cells) compared to standard lipofection (1.31% positive cells using 0.5 μg/150,000 cells) at 48 h. Remarkably, nanoparticles promoted GFP expression in transfected cells at an earlier time after transfection compared to liposomes, since at 16 h, 1.2% cells were GFP-positive in nanoparticle-transfected cells compared to 0.54% in those transfected with liposomes, thus suggesting different cellular uptake and/or intracellular vesicular trafficking routes for the two transfection systems. Quantification on 1100–1600 cells per condition.

Differentiated cells are usually difficult to transfect by standard means even in in vitro cell cultures, since lipofection, electroporation or calcium phosphate-mediated transfection strongly depend on plasma and nuclear membrane composition and physicochemical characteristics, such as lability or stiffness. As a first assay, we attempted to directly compare the transfection efficiency in differentiated retinal pigment epithelium cells using GFP as a reporter gene. Dividing ARPE-19 cells (1.5 × 10^5^ cells) were seeded in 24-well plates. The shift to DMEM minimal medium without supplementation of FBS together with cell confluence promotes differentiation to retinal pigment epithelium cells. Under those conditions, ARPE-19 cells stop dividing and in 48 h produce one cilium per cell, demonstrative of their differentiation state. At that stage, liposome-DNA complexes obtained following manufacturer’s conditions (Lipotransfectine, 0.500 μg per well) and DNA-wrapped gold NPs (40 nm gold nanoparticles from Sigma-Aldrich (St. Louis, MO, USA), wrapped with pEGFP plasmid DNA following the procedure stated in [18]) (0.250 μg per well) were used to transfect ARPE-19 cells. The plasmon resonance of DNA-gold NPs was activated by white light. After 16 h or 48 h post-transfection, fluorescence images were obtained in a ZOE fluorescent cell imager using the same fluorescence settings (Figure 1A,B and Figure S1). Several images were processed per well and condition and the number of green positive cells was counted manually in a total of 1100–1600 cells.

The number of transfected cells, around 1.4%, was very similar using both methods after 48 h (Figure 1C), even though the amount of DNA used was lower (half) for DNA-gold NP complexes. These results indicated that DNA-gold NPs can be used to transfect differentiated cells with a similar yield than other widely used transfection methods. Remarkably, when using DNA-gold NPs, the same percentage of transfected cells (around 1.2%) was detectable at 16 h and at 48 h, which suggested that the expression of the reporter gene was relatively fast (Figure 1C). Liposome-DNA complexes enter through clathrin-coated vesicles and after trafficking through the endosomal pathway mainly end up fusing to lysosomes (where DNA and lipids are degraded) [19,20,21]. Our results showing an early reporter gene expression at 16 h indicated that at least a pool of DNA-gold NP complexes might enter the cell through faster routes than clathrin-coated vesicles, thus escaping the endosomal-lysosomal pathway and reaching the nucleus in less time.

### 3.2. DNA-Gold Nanoparticles Enter RPE Cells by Clathrin-Mediated Vesicles and Other Cellular Uptake Routes

In order to assess whether the cellular uptake of DNA-wrapped gold NPs was clathrin-dependent or independent, we performed colocalization assays of gold NPs and DNA-gold NPs with clathrin-coated vesicles at 2 h post-transfection. Since clathrin internalization is dependent on dynamin activity for closure of the forming vesicles at the membrane, we also treated cells with dynasore for 30 min immediately before transfection. Dynasore is a strong and irreversible inhibitor of dynamin enzymatic activity and therefore, clathrin-coated vesicles can be neither closed up nor internalized. Dynasore also affects the cell membrane stiffness and thus alters lipid raft-mediated processes [22,23].

**Figure 2:**
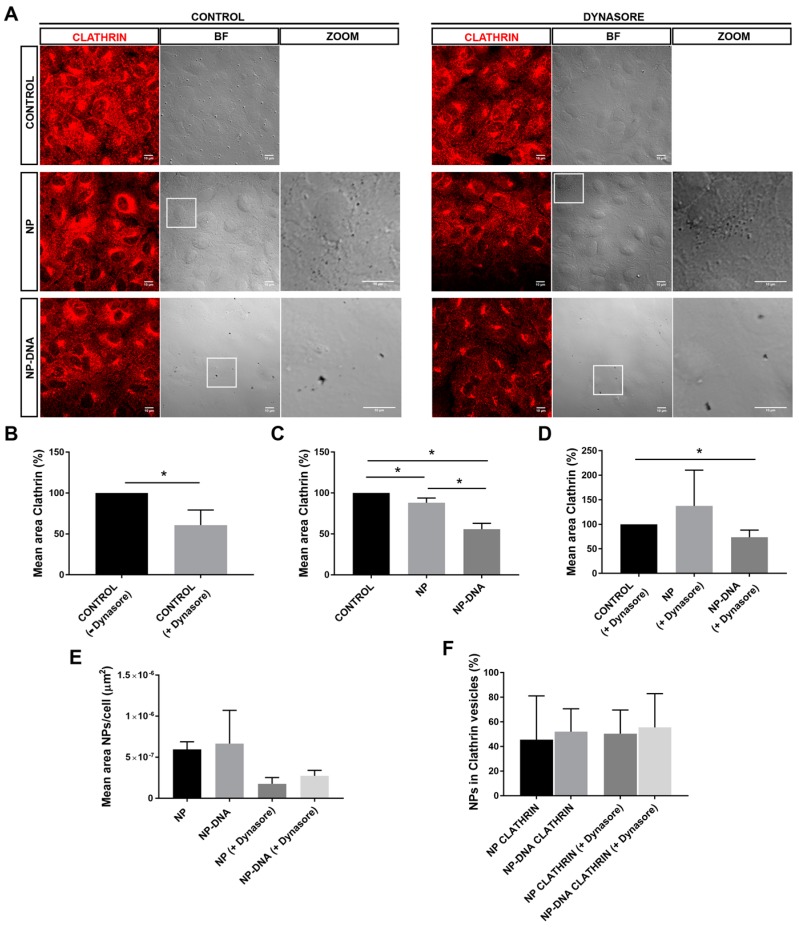
DNA-gold nanoparticles are uptaken by differentiated ARPE-19 cells through different vesicular and/or non-vesicular routes. (**A**) After 2 h post-transfection, between 40 and 60% of pristine gold NPs and DNA-wrapped gold NPs are detected in clathrin-coated vesicles. NPs are visualized as black dots in the optic transmission microscopy channel (BF-bright field, see also zoom panels). DNA nanoparticles appear in clusters compared to pristine 40 nm gold particles. Clathrin-coated vesicles are immunodetected in red. Dynasore treatment inhibited the uptake by dynamin-mediated events. Image analyses of colocalization were performed by using masks over different channels. (**B**) Treatment with dynasore reduced down to 60% the number of clathrin-coated vesicles in untransfected cells, after 2 h. Addition of DNA nanoparticles in either (**C**) control conditions or (**D**) with dynasore treatment significantly reduced down to 55% the number of clathrin-coated particles. (**E**) At 2 h post-transfection, the mean area of NPs/cell uptaken by differentiated RPE is reduced two-fold in dynasore-treated cells, reflecting that at least half of the nanoparticles are internalized by dynamin-mediated events; remarkably, the mean area of internal NPs is higher in cells transfected by DNA-wrapped NPs compared to pristine NPs. (**F**) Percentage of NPs/cell in clathrin-coated vesicles is highly similar in all conditions, ranging between 40 and 50%, indicating that a large pool of NPs was not uptaken by RPE cells using this route. Representative images from three independent replicates (three images per replicate and condition) were quantified. Statistical significance was analysed by the non-parametric Mann–Whitney test (* indicates *p* < 0.05).

Figure 2A shows representative images of the intracellular localization of clathrin vesicles (immunodetected in red) and NPs (detected as black dots in optic tomography field, see zoom panels) in differentiated ARPE-19 cells at 2 h post-transfection. Confocal image stacks were centred in the subcellular localization where clathrin vesicles were more prominent (relatively close to the plasma membrane). Several image masks were used to analyse the localization of NPs in the clathrin-coated vesicles under control (panels at the left) and dynasore treatment (panels at the right) conditions. Inhibition of dynamin-mediated events by addition of dynasore was effective, since it significantly reduced the number of clathrin-coated vesicles down to 60% in control untransfected cells (Figure 2A,B).

Notably, the addition of pristine gold NPs, but particularly of DNA-gold NPs, also significantly reduced the area of clathrin vesicles down to 55% compared to control untransfected cells, which were considered the control reference (Figure 2A panels at the left, and Figure 2C), in a reduction effect similar to that caused by dynasore treatment. This reduction in the area of clathrin vesicles in cells transfected with DNA-gold NPs was also observed after treatment with dynasore (Figure 2A panels at the right, and Figure 2D). This consistent reduction in clathrin area (in all cases after correction per number of cells in each image) may reflect that at the cell–NP interface, surface properties of the DNA-gold NPs, which also differ from those of pristine gold NPs, can alter the formation in number and/or size of clathrin vesicles at the cell membrane of ARPE-19 cells.

Cell uptake of either pristine gold NPs or DNA-gold NPs (measured by the mean area covered by black dots in each image corrected per cell) was reduced, but not abrogated, after dynamin-inhibition in both cases (Figure 2E). However, the percentage of NPs colocalizing within clathrin-coated vesicles was maintained in all conditions and for both types of NPs, with no statistically significant differences (Figure 2F). Therefore, although dynasore treatment reduced the number of clathrin vesicles as well as the total amount of internalized NPs per cell, the percentage of NP uptake via clathrin by ARPE-19 cells was maintained close to 50%.

Overall, these results showed that the presence of DNA-gold NPs (40 nm diameter) altered the dynamics of the differentiated ARPE-19 plasma membrane since the area of clathrin-coated vesicles was diminished at 2 h post-transfection. The percentage of gold NPs in clathrin-coated vesicles was around 50% in all tested conditions, irrespective of the presence of the DNA wrapping and despite inhibition of dynamin activity. Therefore, at least 50% of gold NPs (40 nm), with or without DNA, could enter differentiating ARPE-19 cells through non-clathrin-mediated means.

### 3.3. Differences in the Intracellular Endosomal Trafficking of DNA-Wrapped Gold NPs Compared to Pristine Gold NPs in RPE Cells

**Figure 3:**
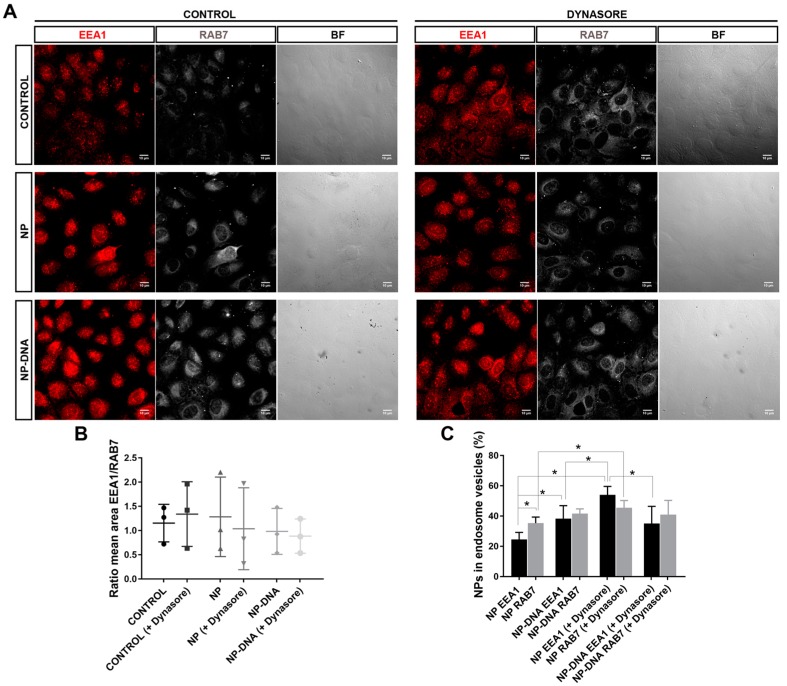
A significant pool of DNA-wrapped gold NPs colocalized in early and late endosomes in differentiated ARPE-19 cells. (**A**) After 2 h post-transfection, a significant pool of NPs colocalized in early endosomes (early endosome marker EEA1, in red) and late endosomes (mature endosome marker RAB7, in white). NPs are visualized as black dots in the optic transmission microscopy channel (BF). Dynasore treatment (panels at the right) was also performed to inhibit dynamin-mediated uptake events. The merge field is not shown since nanoparticles were not distinguishable on the dark background of immunofluorescent images, but image analyses of colocalization were performed by using masks over different channels. (**B**) The number of early and late endosomes was analysed as a ratio (to normalize per cell and condition). After 2 h of transfection with NPs, the ratio EEA1/RAB7 was variable but not significantly different either between cells treated with dynasore compared to controls or between control cells compared to cells transfected with pristine gold NPs or DNA nanoparticles. (**C**) Percentage of pristine NPs and DNA–nanoparticle colocalization with early or late endosomes/cell. At 2 h post-transfection, NPs showed a fluid trafficking between early and late endosomal compartments, as detected by 35% localization in late (35%) versus 25% in early endosomes. Instead, DNA NPs showed a higher localization in the endosomal compartment (close to 100%) but with a similar distribution between early and late endosomes. The addition of dynasore changed the proportion of NPs detected in early and late endosomes when analysing pristine NPs, but not when analysing DNA NPs, indicating that internalization and intracellular trafficking routes differ between pristine gold NPs and DNA NPs. Representative images from three independent replicates (three images per replicate and condition) were quantified. Statistical significance was analysed by the non-parametric Mann–Whitney test (* indicates *p* < 0.05).

Once inside the cell, clathrin-coated and other endocytic vesicles (e.g., receptor- or caveolin-mediated) enter into the dynamics of the endosomal pathway. The key internalization proteins (clathrin, receptors, caveolin) are recycled and the vesicles fuse to endosomes. Early endosomes mature into late endosomes, and this maturation process involves shifts in the protein epitopes of the endosomal membranes. Late endosomes usually fuse to lysosomes, which degrade the engulfed particles within. There are some exceptions to this endosomal trafficking pathway that leads to degradation. For instance, caveolin-mediated vesicles fuse to neutral endosomes and particles within can be liberated in the cytoplasm and bypass degradation [7,14]. Therefore, we decided to analyse the colocalization of internalized gold NPs with endosomal markers, to assess whether NPs were localized in early and/or late endosomes. After 2 h post-transfection with either pristine gold NPs or DNA-gold NPs, under control and dynamin inhibition conditions, differentiated ARPE-19 were immunodetected for EEA1 (in red) and RAB7 (in white), which are markers for early and late endosomes (Figure 3A). Confocal image stacks were selected at the internal subcellular region with an optimum detection of early and late endosomes. NPs were visualized as black dots in optic transmission microscopy. Several masks were applied to quantify mean areas of NPs and colocalization of NPs within early or late endosomes.

First, we assessed that the ratio of early and late endosomes did not vary comparing control cells with cells transfected with either pristine gold NPs or DNA-gold NPs after 2 h post-transfection, in either normal conditions or post-treatment with dynasore (when applicable). No significant differences between the ratio of areas of total early/late endosomes per cell were observed in any tested condition and transfection, including cells treated with dynasore (Figure 3B). However, a more accurate quantification to assess the percentage of NPs within each endosomal compartment, whether in early or in late endosomes, did show statistically significant differences (Figure 3C) when using different NPs and conditions.

Concerning pristine gold NPs, 35% localized in late endosomes and only 25% within the early endosome compartment (Figure 3C). Therefore, the amount of gold NPs that did not localize to the endosomal pathway was close to 40%. In clear contrast, DNA-gold NPs showed an equivalent distribution in both compartments, early and late, summing up to a total of 80% of NPs within the analysed endosomal compartments and 20% outside endosomes. These difference in distribution between pristine and DNA-gold NPs was statistically significant (Figure 3C).

On the other hand, the NPs trafficking through the endosomal pathway after inhibiting dynamin was also divergent between the two types of NPs. After dynasore treatment, pristine gold NPs were mainly found in the endosomal compartments (summing up to 100%), with a significant high increase in the localization within the early endosomes compared to control conditions. Cells transfected with DNA-gold NPs responded differently, since there was no significant variation between NPs localizing at early or late endosomes, rendering similar values to untreated cells, overall indicating that a pool of DNA-gold NPs did not enter through clathrin-coated vesicles. The high number of early endosomes with DNA-wrapped NPs in treated and untreated cells suggest that these particles promote an endocytosis route with a higher number of early endosomes that do not mature into the late compartment. These endosomes might release the cargo into the cytoplasm (in this case, the reporter plasmid pEGFP), which could reach the nucleus and be expressed much faster compared to the early/late endosomal trafficking followed by liposomes, as observed by the early GFP detection at 16 h when using DNA-gold NPs.

## 4. Discussion

Gene augmentation therapy in recessive mendelian disorders caused by loss of function mutations is, in principle, a plausible option for treatment. Addition of a wild-type copy of the gene should be effective, provided that the gene is delivered and correctly expressed at the target tissue. In IRDs, retinal degeneration is caused by mutations in either genes expressed in photoreceptors or genes expressed in the adjacent RPE, as it is the case with mutations in RPE65, MERTK or LRAT. AAV-based vectors are being used for gene delivery to photoreceptors, but non-viral vectors based on NPs should be concurrently explored for cases in which viral approaches may not be desirable [3,4,8]. Several reports have shown that RPE is particularly amenable for transfection with NPs. This apparent feasibility may be due to the phagocytic nature of RPE cells, which easily engulf and internalize photoreceptor outer segments as well as other exogenous particles [4,9]. Phagocytosis involves the formation of large vesicles, but most small particles enter the cell via pinocytosis, which involve internalization routes mediated by different protein and lipid interactions, for example, clathrin- and caveolae-mediated vesicles as well as clathrin- and caveolae-independent endocytosis [7,14]. The endocytic entry route is key to the final outcome of the internalized particles, since cargo internalized via clathrin vesicles mainly end up being degraded by the lysosome, whereas cargo internalized by caveolin-mediated vesicles or clathrin-independent routes bypass lysosomal degradation. In this context, we have explored the potential use of gold NPs to transfect RPE cells in culture, focusing on the uptake routes and the intracellular endosomal trafficking pathways.

Several authors have reported that in vitro biocompatibility of gold nanoparticles largely depended on their shape and size, and determined that sizes between 5 and 30 nm were more cytotoxic, even though the rate of internalization was higher than that of the less toxic NPs of 50–100 nm (reviewed in [10]). We have used NPs of 40 nm as a compromise between cytotoxicity and internalization. In addition, surface chemical modification of NPs is a critical step that decreases toxicity, increases stability, reduces aggregation and modulates cellular uptake. Most NPs use positive charges at the surface to facilitate the entry at the negatively charged cell membranes, but positively charged NPs induce cell death, whereas negatively charged surfaces induce internalization by clathrin/caveolae-independent endocytosis [7,14]. In this context, we have used plasmid DNA wrapping over gold NPs for two different but relevant reasons: as a means to stabilize gold NPs [8] and favour clathrin-independent cellular uptake, as well as to provide the DNA molecule to be delivered.

In differentiated ARPE-19 cells, at least up to 48 h post-transfection, DNA-wrapped gold NPs did not show any toxicity. DAPI staining did not detect any pyknotic cells, and the transfection efficiency was equivalent for both systems (Figure 1 and Figure S1), even though NPs required half the amount of DNA for the same rate of transfection, thus indicating that this point could be further optimized in the future. Interestingly, in cells transfected with DNA-gold NPs, the same number of cells expressed the reporter GFP gene at 16 h as at 48 h, which suggested that DNA-gold NPs might use a faster uptake/internalization route than cationic liposome-DNA complexes, which are mostly internalized via clathrin vesicles [14,19,20,21].

Our assays also determined that, in vitro, RPE cells responded differently to pristine gold NPs and DNA-gold NPs, since the area of clathrin vesicles was reduced in the presence of DNA-gold NPs. These differences might be due to changes in the membrane–NP interface. On the one hand, at least 50% of the pool of pristine gold NPs and DNA-gold NPs were internalized via clathrin-coated vesicles, which most probably ended up in the endo-lysosomal degradation pathway (Figure 2). It is likely that this 50% of 40 nm NPs internalized by clathrin-dependent endocytosis corresponded to soluble NPs. On the other hand, the other half of the pool of pristine gold NPs and DNA-gold NPs uses different alternative endocytic routes for internalization, as shown by different localization/trafficking to endosomal compartments. At least 30% of pristine gold NPs are internalized by non-vesicular routes and localized neither in early nor in late endosomes. Since after dynasore treatment this alternative entry route was inhibited and all pristine NPs were detected in the endosomal pathway, the internalization of a significant pool of gold NPs was through lipid rafts or diffusion by destabilization of the cell membrane [22,23]. In contrast, the pool of DNA-gold NPs that was not detected in the endosomal pathway was much lower. A preference for vesicular entry could be explained by the larger size of the DNA-gold NP complexes. Interestingly, DNA-gold NPs are equivalently distributed in early and late endosomes, irrespective of dynasore treatment, thus indicating that a pool of DNA-gold NPs are found in early endosomes that will not mature into late endosome vesicles. These results are in agreement with the earlier expression of the reporter gene detected at 16 h post-transfection, which point to a pool of DNA-gold NPs being internalized by clathrin-independent endocytosis (for instance, via caveolin-mediated vesicles [7,14]) and to cargo escaping lysosomal degradation. Nonetheless, we cannot discard the result that DNA-gold NPs used alternative endosomal trafficking or recycling routes [24].

Our results are but a first step towards the potential use of non-cytotoxic gold NPs for in vivo gene therapy in retinal disorders. Indeed, several basic biological questions should be first addressed, such as the internalization routes and their intracellular endosome trafficking. According to our initial results, DNA-wrapped gold NPs might be considered for gene delivery in retinal tissues. However, before considering this system for in vivo applications, further work is required to fully understand the complexity of cell–NP interaction, which is crucial to increase their transfection efficiency, optimize the cargo delivery by avoiding lysosomal, autophagic or other degradative pathways, and ensure sustained expression of the therapeutic gene.

## 5. Patents

S.T. declares that the DNA wrapping protocol on NPs is unpublished and currently subject to a patent (S. Trigueros Great Britain Patent GB201201207484A and United States Patent Application 20180318424).

## Supplementary Materials

The following are available online at https://www.mdpi.com/2073-4425/10/4/289/s1, Figure S1. Wider field image of Figure 1, showing transfection efficiency of NPs in ARPE-19 cells.
