# Supplementary material for: In Vitro Gene Delivery in Retinal Pigment Epithelium Cells by Plasmid DNA-Wrapped Gold Nanoparticles
Source: Genes (Basel). 2019 Apr 9;10(4):289. doi: 10.3390/genes10040289 (PMC6523520; doi:10.3390/genes10040289)
Supplement: Supplementary file 1 [file genes-10-00289-s001.pdf]

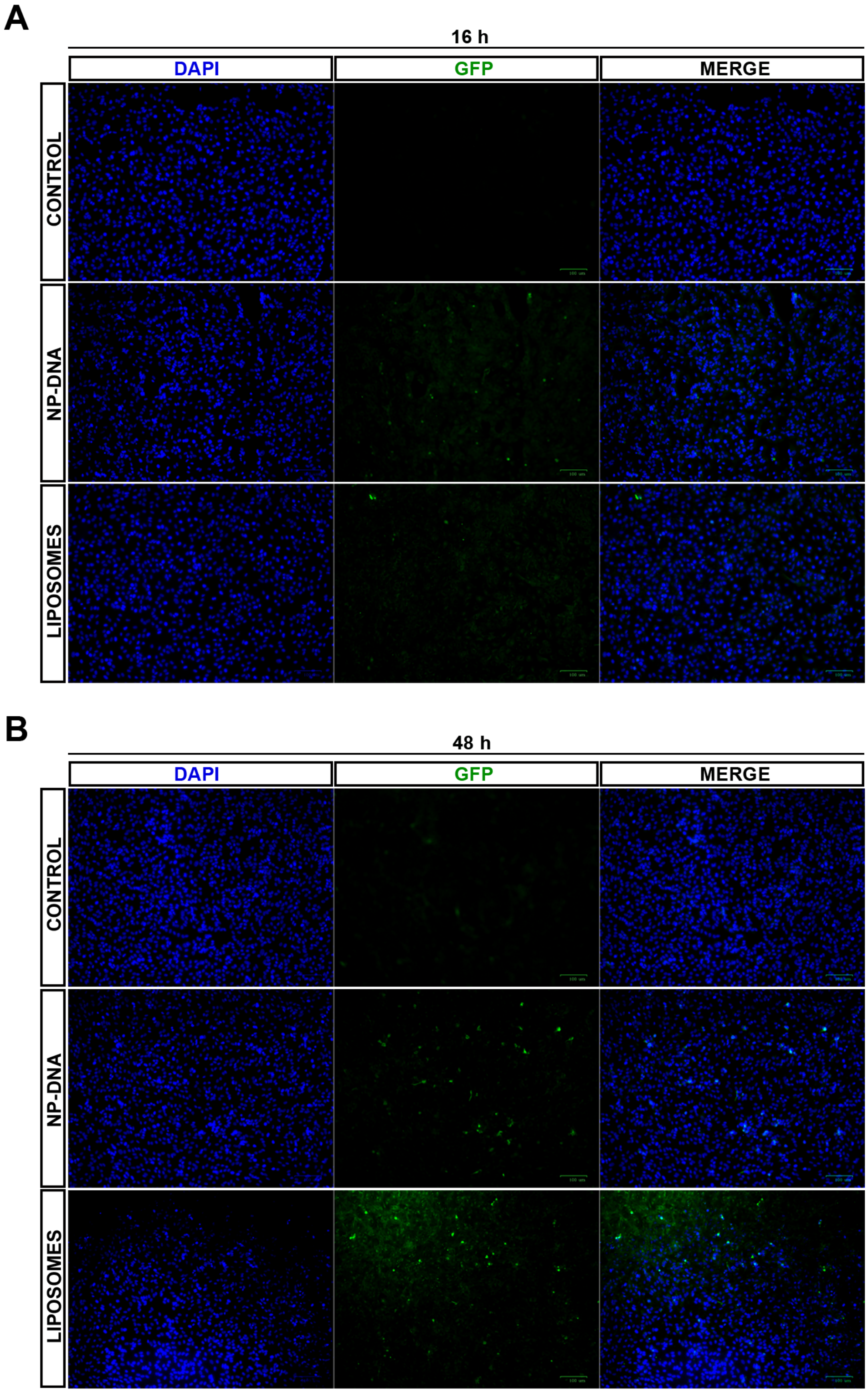

Supplementary Figure 1.- Transfection efficiency of DNA wrapped-gold nanosomes (40 nm) compared to liposomes in differentiated ARPE-19 cells (wider field image of Figure 1)**.** Representative image of differentiated ARPE-19 cells transfected with the pEGFP reporter vector using either liposomes (LIPOTRANSFECTINE) or nanosomes (DNA-NPs) at 16 hours **(A)** and 48 hours **(B)**, showing that a higher number of cells were GFP positive at 16 h in nanosome- versus liposome-transfected cells, indicating differences in the cellular uptake and/or intracellular vesicular trafficking routes between the two transfection systems (Images obtained in a ZOE™ Fluorescent Cell Imager).
